# Diagnostic accuracy and efficiency of combined acquisition of low-dose time-resolved and single-phase high-resolution contrast-enhanced magnetic resonance angiography in a single session for pre-angiographic evaluation of spinal vascular disease

**DOI:** 10.1371/journal.pone.0214289

**Published:** 2019-03-28

**Authors:** Jae Ho Shin, Yangsean Choi, Borim Park, Na-Young Shin, Jinhee Jang, Hyun Seok Choi, So Lyung Jung, Kookjin Ahn, Bum-soo Kim

**Affiliations:** Department of Radiology, Seoul St. Mary’s Hospital, The Catholic University of Korea, Seoul, Korea; Jagiellonian University Medical College, POLAND

## Abstract

**Background/Purpose:**

The purpose of this study was to evaluate the utility and efficacy of combined low-dose, time-resolved (TR) and single-phase high-resolution (HR) contrast-enhanced MRA (CE-MRA) as a pre-angiographic study for spinal vascular disease.

**Materials and methods:**

Seventeen consecutive patients with suspected spinal vascular disease were retrospectively reviewed. All patients underwent combined low-dose TR CE-MRA and three-dimensional single-phase HR CE-MRA at 3 Tesla, followed by conventional spinal digital subtraction angiography (DSA) within 90 days. Six patients underwent additional spinal MRA and DSA for treatment follow-up. Spinal lesions were analyzed in terms of presence, disease type, laterality, spinal level, and number of arterial feeders.

**Results:**

Low-dose TR CE-MRA helped proper localization of subsequent HR CE-MRA in two patients with high or low level of the lesion. For initial detection of spinal vascular disease, sensitivity, specificity and accuracy of CE-MRA were 93.3% (n = 14/15), 100% (n = 3/3), and 94.4% (n = 17/18), respectively. In characterization of dural arteriovenous fistula (AVF), perimedullary AVF, spinal cord arteriovenous malformation (AVM), and extraspinal AVM, CE-MRA correctly characterized in 86.7% (n = 13/15) among the positive findings, and in 88.9% (n = 16/18) among the several patients including negative results. CE-MRA showed matched per-case localization of arterial feeders within 1 vertebral level in 80% (n = 12/15), and matched per-lesion localization in 78.3% (n = 18/23).

**Conclusion:**

Combined low-dose TR CE-MRA and single-phase HR CE-MRA at 3 Tesla was an effective and accurate non-invasive tool for the pre-angiographic evaluation of spinal vascular diseases in a single session.

## Introduction

Spinal digital subtraction angiography (DSA) remains the gold standard for diagnosing, localizing, and classifying spinal vascular diseases [1]. Despite the superior diagnostic value of DSA, it is an invasive, time-consuming procedure, with associated risks for ionizing radiation exposure, and procedure-related complications such as dissection and thrombosis formation [2–5]. The limited field of view (FOV) of spinal MRA, considering the long spinal axis, makes detection in a single MRA session difficult [6–10]. The use of 3T MR considerably improves image quality, in addition to a reduction in acquisition time. Specifically, spinal dynamic contrast-enhanced MRA (CE-MRA) at 3T enables assessment of spinal vascular disease, as well as visualization of arterial and venous separation [6, 11–13]. Furthermore, administration of low-dose contrast is feasible in time-resolved (TR) CE-MRA, which enables rapid acquisition of important functional information for the evaluation of normal and pathological vascular structures [14, 15]. Low-dose TR CE-MRA is also useful for detecting bolus arrival at the region of interest, thus facilitating the acquisition of subsequent single-phase high-resolution (HR) CE-MRA. Taking advantage of this combined acquisition of low-dose TR CE-MRA and single-phase HR CE-MRA at 3T, pre-angiographic evaluation of spinal vascular diseases can be achieved. The purpose of this study, therefore, was to evaluate the feasibility and diagnostic utility of combined low-dose TR and single-phase HR CE-MRA as a pre-angiographic study in patients with spinal vascular disease.

## Materials and methods

The present study was approved by the institutional review board, and a waiver of consent was obtained for a Health Insurance Portability and Accountability Act-compliant retrospective study, by our institution IRB (Catholic Medical Center Institutional Review Board). Seventeen consecutive patients (12 male, 5 female) with suspected spinal vascular disease were investigated at a single institution between March 2009 and June 2018. Our patient inclusion criteria were patients above 18 years of age and high clinical suspicion of spinal vascular diseases (e.g. leg weakness, paresthesia, and incontinence). Patients with any other underlying disease, such as vasculitis, hypervascular tumor or malignancy, were excluded from the study.

### MR protocol

MRA was performed using a 3T whole-body MR scanner (Magnetom Verio, Siemens Healthcare, Erlangen, Germany) to obtain low-dose TR CE-MRA and sequential three-dimensional (3D) single-phase HR CE-MRA. Low-dose TR CE-MRA was initially performed to aid bolus-timing as well as proper localization for the subsequent 3D single-phase HR CE-MRA.

Low-dose TR CE-MRA using time-resolved angiography with interleaved stochastic trajectories (TWIST) was performed in the sagittal plane after a standard automated bolus intravenous injection (Spectris, Medrad, Pittsburgh, PA, USA) of 0.03 mmol/kg body weight of gadobutrol (Gadovist, Bayer healthcare, Germany) at a flow rate of 1.5 mL/s, followed by 20 mL of saline flush at the same rate, using 20-gauge needle. Imaging parameters were as follows: repetition time, 2.95 ms; echo time, 1.08 ms; FA, 18°; FOV, 280 × 262 mm; matrix, 320 × 170; bandwidth, 600 Hz; GRAPPA factor 2; and voxel size, 0.9 × 1.7 × 1.2 mm^3^. The percentage of k-space center volumes were 10% and percentage of k-space periphery volumes were 20%. TWIST sequence and k-space parameters have been presented in detail previously [14, 15]. Having set the k-space parameters, TWIST sequence was used for spinal low-dose TR CE-MRA, which provided a temporal resolution of 2.81 s after temporal interpolation.

The 3D single-phase HR CE-MRA was based on a sagittal 3D gradient echo sequence using the following parameters: repetition time, 3.43 ms; echo time, 1.33 ms; FA, 25°; FOV, 280 × 166 mm; matrix, 384 × 269; bandwidth, 650 Hz; voxel size, 0.7 × 1.0 × 0.5 mm^3^; and acquisition time, 1 min 16 s. A standard automated bolus injection of 0.1 mmol/kg body weight gadobutrol was, once again, used at a rate of 1.5 mL/s, followed by a 20 mL saline flush injected at the same rate. The images were acquired 4 s after contrast arrival at the aorta (T10 level in most cases, but could be adjusted by suspected lesion level illustrated on the low-dose TR CE-MRA). The 4 s delay was used for better visualization of spinal DAVF, as described by Farb et al. [8] because small spinal DAVFs are generally small slow-flow fistulas that require time to fill from the feeding artery to the perimedullary vein. Automated maximum intensity projection (MIP) or volume-rendering reconstructions were generated for the entire FOV. Segmented MIP images were also acquired in standard planes (coronal, sagittal, and axial).

### DSA technique

All patients underwent DSA examinations for the diagnosis of spinal vascular disease within 90 days of MRA. Selective spinal DSA was performed via a transfemoral approach with the patients under local anesthesia. The pre-angiographic CE-MRA facilitates identifaction of the origin sites of each intercostal and lumbar arteries from the aorta, and the possible hypoplasia of a certain segmental artery as well. Therefore, the duration of spinal DSA in the authors’ institute was mostly within an hour, and most patients could tolerate the procedure and were cooperative during angiographic data acquisition under the local anesthesia. Imaging acquisition was in the frontal projection at 3 frames/s. Standardized angiography included selective manual injections of 2–3 mL of the nonionic contrast agent iodixanol (Visipaque 270 [270 mg iodine/mL], GE Healthcare, Cork, Ireland) into the lumbar and intercostal arteries. When necessary, additional injections were made into median sacral artery, bilateral iliolumbar arteries, supreme intercostal arteries, costocervical trunks, thyrocervical trunks, external carotid arteries, and vertebral arteries to evaluate the possible feeders.

### Image evaluation

Images were analyzed by two experienced radiologists (B.K. and K.A.), who had experience of neuroradiology more than 10 years and they were aware that the MR examinations were performed in patients with suspected spinal vascular disease. Patients’ data such as clinical history, findings and the results of DSA were blinded. Both observers independently analyzed the MRA examinations to assess the type of spinal vascular disease, laterality, vascular lesion level, and number of arterial feeders; any disagreements were resolved by consensus. For the MRA evaluation, arteriovenous fistulas (AVFs) were suspected when the following features were present: early visualization of perimedullary vein; prominent radicular artery; and prominent draining veins. Lesion type was also based on the locations of the fistulas such as the surface of dura (dural AVF) and spinal cord (perimedullary AVF), and outside the spinal canal (extraspinal AVF). An arteriovenous malformation (AVM) was suspected when nidus formation with engorged perimedullary venous plexus or draining veins were noted. All DSA images were also evaluated by the two observers, both of whom were blinded to the results of the MRA examinations. The type of spinal vascular disease, and the exact location, laterality, origin, and number of arterial feeders were evaluated; any disagreements were resolved by consensus.

## Results

A total of 17 patients (5 female, 12 male) were enrolled in the study. The mean patient age was 42 years (range, 18–74 years). The average age of the male patients was 51 years (range, 18–74 years), while the average age of the female patients was 42 years (range, 23–75 years). For all patients, a total of 24 MR imaging evaluations (18 initial MRA and 6 post-treatment follow-ups) were performed, which were followed by DSA. The interval time between CE-MRA and subsequent DSA examination was 12 days. A patient, in whom perimedullary AVF (case 7 in patient 6) had been surgically excised, presented a new metachronous spinal dural AVF (case 20 in patient 6) on follow-up CE-MRA and DSA in 5 years later. Therefore, these findings were included as initial evaluations of new metachronous vascular lesions. Patients and their diagnostic results on spinal CE-MRA and DSA are summarized in Table 1.

**Table 1:** Summary of patients and findings of contrast enhanced MR angiography and DSA.

Case	Patient	Sex	Age	Study	CE-MRA		DSA	
					Diagnosis	Feeder	Diagnosis	Feeder
1	1	M	53	Initial	dAVF	Lt. T7	dAVF	Lt. T7
2	2	F	48	Initial	scAVM	Lt. T9	pmAVF	Lt. T8
3	3	M	54	Initial	dAVF	Rt. T12	dAVF	Rt. T12
4	4	M	54	Initial	dAVF	Lt. S2	dAVF	Lt. S2
5	3			FU	no lesion		no lesion	
6	5	M	31	Initial	scAVM	Rt. T10, Lt. T10, Lt. T9	scAVM	Rt. T10, Lt. T10, Lt. T9
7	6	F	33	Initial	pmAVF	Lt. L3	pmAVF	Lt. L3
8	7	M	63	Initial	dAVF	Rt. L1	dAVF	Rt. L1
9	8	F	74	Initial	no lesion		ExSpAVM	Lt. L4, Lt. L3
10	8			FU	no lesion		ExSpAVM	Lt. L3
11	9	M	40	Initial	scAVM	Lt. T9	scAVM	Lt. T9
12	10	M	23	Initial	pmAVF	Rt. T8, Lt. T8	pmAVF	Rt. T12, Rt. T8, Lt. T8, Lt. L1
13	10			FU	no lesion		no lesion	
14	11	M	23	Initial	no lesion		no lesion	
15	12	M	65	Initial	dAVF	Lt. T5	dAVF	Lt. T5
16	13	F	18	Initial	pmAVF	Lt. L1, Lt. L2	pmAVF	Lt. L1, Rt. T10
17	12			FU	no lesion		no lesion	
18	13			FU	no lesion		no lesion	
19	14	M	64	Initial	dAVF	Rt. T12, Lt. L1	dAVF	Rt. T12, Lt. L1
20	6			Initial*	dAVF	Rt. S2	dAVF	Rt. S2
21	15	M	75	Initial	no lesion		no lesion	
22	12			FU	no lesion		no lesion	
23	16	F	36	Initial	no lesion		no lesion	
24	17	M	61	Initial	dAVF	Rt. T6	dAVF	Rt. T6

*a new metachronous lesion on follow-up evaluation

Abbreviations: Lt: Left. Rt: Right, dAVF: dural arteriovenous fistula, scAVM: spinal cord arteriovenous malformation, pmAVF: perimedullary arteriovenous fistula, ExSpAVM: extraspinal arteriovenous malformation, FU: follow-up

**Fig 1:**
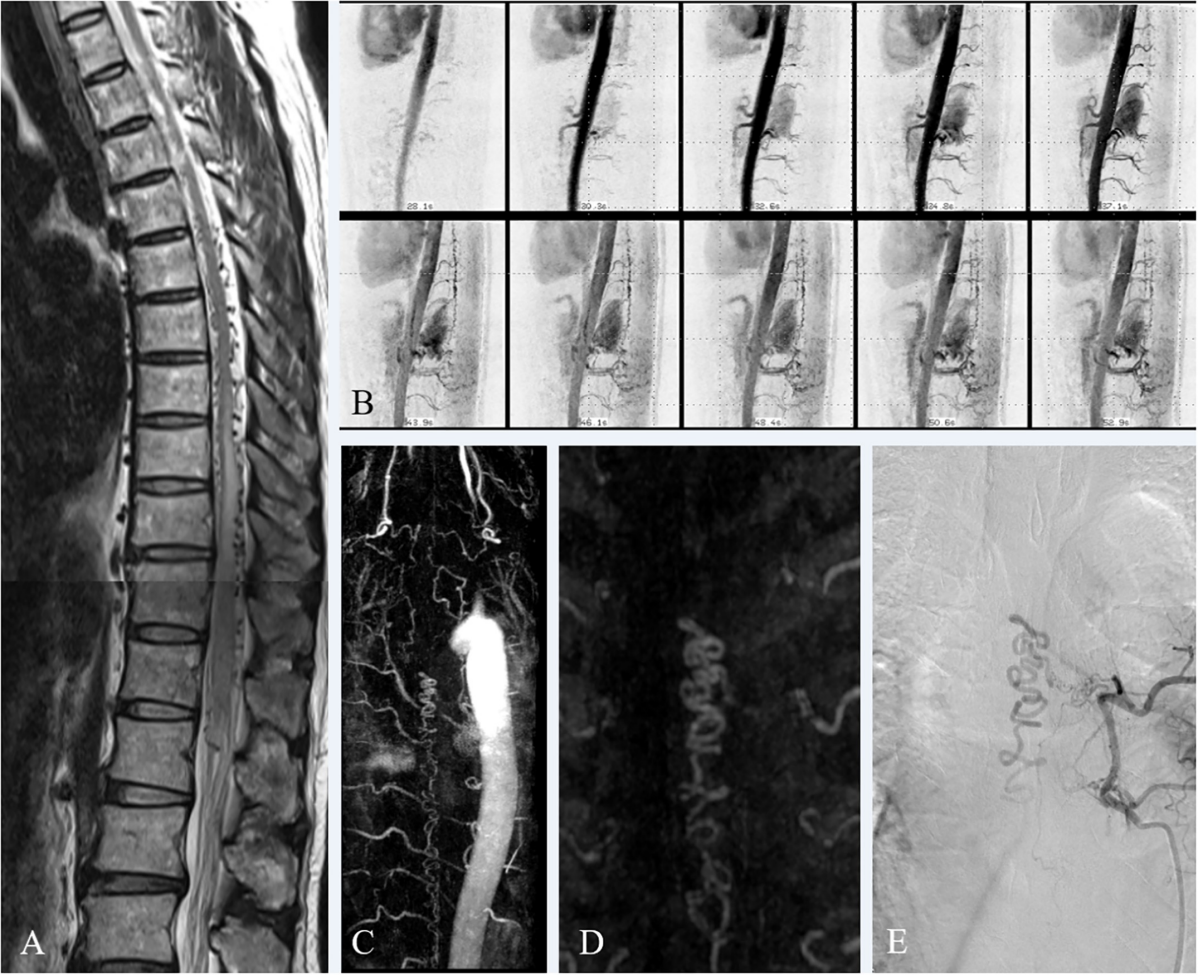
A 53-year-old man with spinal dural arteriovenous fistula (DAVF) (patient 1). T2-weighted sagittal image (A) revealing tortuously dilated perimedullary veins in the thoracic and lumbar spinal canal. Serial maximum intensity projection (MIP) images of low-dose time-resolved contrast-enhanced magnetic resonance angiography (CE-MRA) (B) revealing sequential visualization of dilated perimedullary veins from the top of the field-of-view. MIP images of the entire field of view (C) and coronal segmental MIP image (D) of three-dimensional single-phase high-resolution CE-MRA revealed fistula at the left T6 level. Spinal digital subtraction angiography (E) confirmed spinal DAVF with shunt located at the matched level.

Low-dose TR CE-MRA enabled sequential visualization of arterial- and venous-phase images of MRA in all patients, as well as the venous drainage pattern of the spinal vascular disease. In 2 of 18 (11.1%) initial evaluations, low-dose TR CE-MRA helped proper localization of subsequent HR CE-MRA. In patient 1, TR CE-MRA with the FOV located at the lower thoracic and lumbar regions revealed sequential visualization of the engorged perimedullary vein in the craniocaudal direction from the top (possibly from cranially beyond the FOV), which suggested that the shunt was cranial to the FOV. Therefore, subsequent single-phase HR CE-MRA was performed with the FOV including the upper thoracic spine and revealed a spinal DAVF with an arterial feeder originating from the left T6 intercostal artery, which was confirmed by DSA (Fig 1). Similarly, in patient 4, low-dose TR CE-MRA revealed sequential visualization of the engorged perimedullary vein in the caudocranial direction from the bottom of FOV. Thus, single-phase HR CE-MRA was performed with the FOV including the aortoiliac vessels, which demonstrated a spinal dural AVF from the S2 branch of the left internal iliac artery. The lesion and its feeding vessels were also confirmed by DSA.

**Fig 2:**
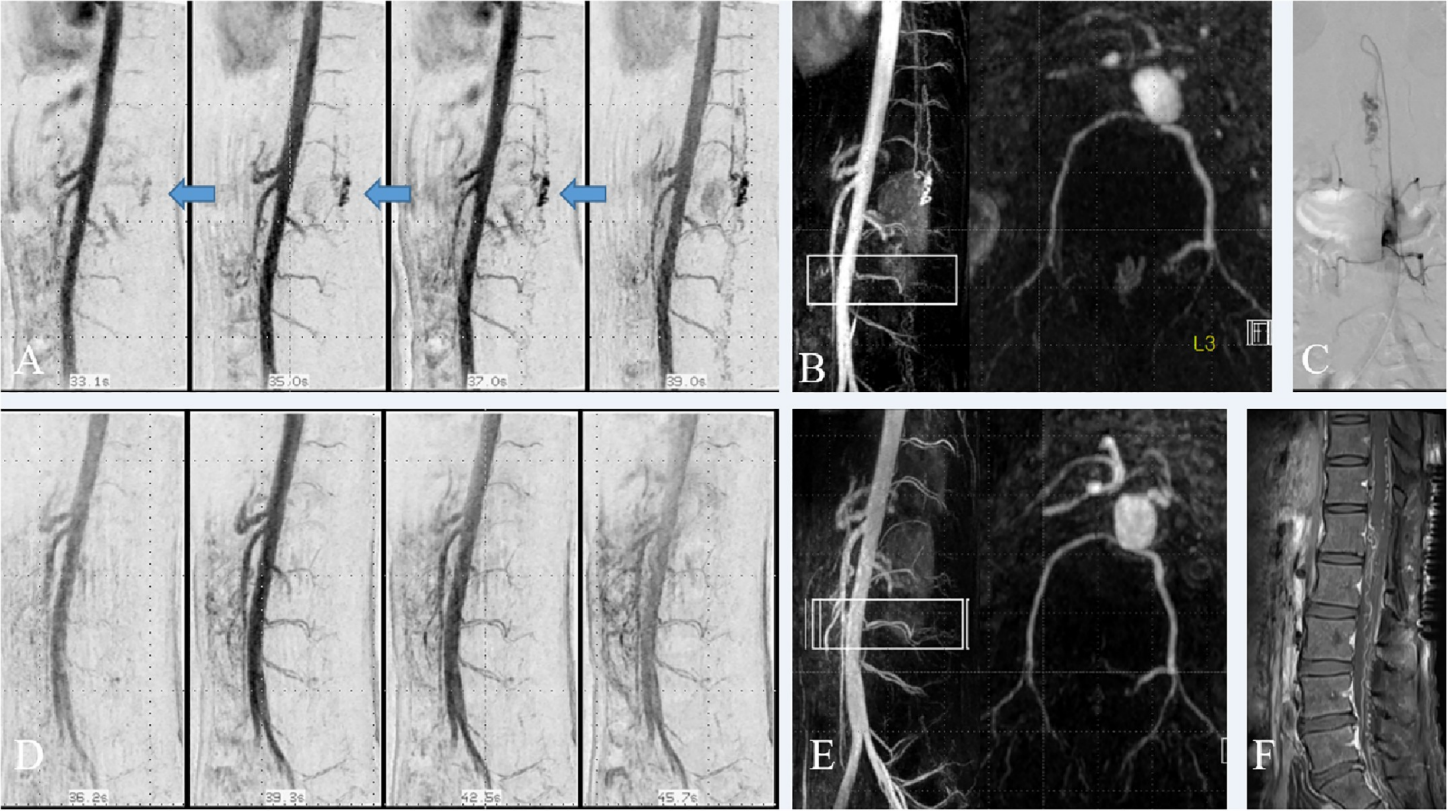
A 33-year-old man with perimedullary arteriovenous fistula (pmAVF) (patient 6). Sequential MIP images of low-dose TR CE-MRA showed early visualization of dilated venous sac (arrow) and perimedullary venous drainage (A). In addition, there were hypoplastic both 1st lumbar arteries, which were confirmed on subsequent DSA (not shown). MIP and segmented axial MIP images of single-phase HR CE-MRA (B) showed spinal AVM with feeding artery of anterior spinal artery from prominent radicular artery at left L3 level. Spinal DSA (C) confirmed spinal AVM with anterior spinal artery arising at the matched level. Follow up low-dose TR CE-MRA (D) obtained 7 days after surgery revealed obliteration of previously noted dilated venous sac. MIP and segmented axial MIP images of follow up single-phase HR CE-MRA (E) also showed obliteration of dilated venous sac, yet with still prominent radicular artery at left L3 level and several perimedullary vessels. Delayed contrast enhanced T1 weighted MR image (F) also showed contrast enhancement at the perimedullary vessels.

**Fig 3:**
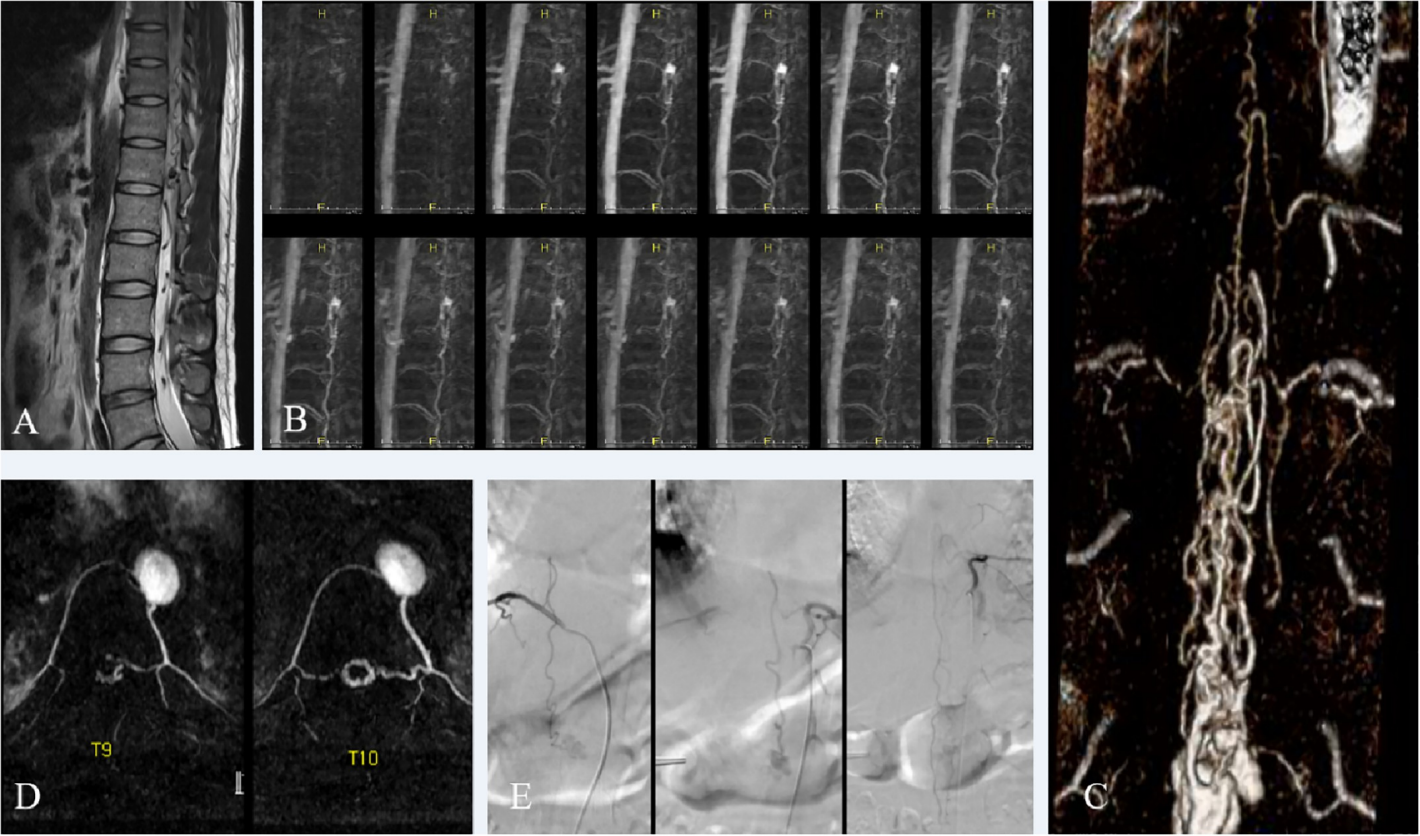
A 31-year-old man with spinal arteriovenous malformation (AVM) (patient 5). Sagittal T2-weighted image (A) revealed tortuous vascular signal voids. Serial maximum intensity projection (MIP) images of low-dose time-resolved contrast-enhanced magnetic resonance angiography (CE-MRA) (B) revealed early visualization of a dilated venous sac. Volume-rendering reconstruction image (C) and segmental MIP images (D) of single-phase high-resolution CE-MRA revealed arterial feeders of the shunt, including the anterior spinal artery arising from the left T9 intercostal artery and posterior spinal arteries from both T9 intercostal arteries. Spinal digital subtraction angiography (E) confirmed spinal AVM with matched level of feeding arteries.

Among the 18 initial evaluations, pre-angiographic CEMRA detected 14 spinal vascular lesions including spinal cord AVMs (n = 3), perimedullary AVFs (n = 3), dural AVFs (n = 8), while 4 subjects came out as negative findings. The spinal DSA confirmed 15 spinal vascular lesions including spinal cord AVMs (n = 2), perimedullary AVFs (n = 4), dural AVFs (n = 8), extraspinal AVM with intradural venous drainage (n = 1), and 3 subjects came out as negative findings (Table 2). It is possible that these 3 negative subjects may have tiny, inconspicuous vascular lesions that we have overlooked. However, after careful reviews and clinical correlation, we confirmed that three negative subjects did not have tiny vascular malformations. In comparison with DSA findings, CE-MRA corrected diagnosed 16 out of 18 cases; 3 negative findings on DSA, 8 dural AVFs (Fig 1), 3 of 4 perimedullary AVFs (Fig 2), and 2 spinal cord AVMs (Fig 3). CE-MRA showed negative findings in a patient (case 9) with extraspinal AVM in pelvic cavity with spinal intradural venous drainage on DSA. In a patient (case 2) with perimedullary AVF on DSA, CE-MRA suggested spinal cord AVM. Therefore, as initial evaluation of the spinal vascular lesions, the CEMRA demonstrated superb sensitivity (93.3%; 95% CI: 68.1%–99.8%), specificity (100%; 95% CI: 68.1%-99.8%) and accuracy (94.4%; 95% CI: 72.7%–99.8%) in detecting the presence of lesions. In terms of disease characterization, CE-MRA was able to correctly distinguish spinal vascular disease in 86.7% (13 of 15) among the positive findings. When patients without spinal vascular disease were taken into account, matched disease characterization was 88.9% (16 of 18) (Table 3). In the initial evaluation for the per-case localization of arterial feeders within 1 vertebral level, CE-MRAs detected 12 matched cases out of 15 cases with spinal vascular disease (80%). For the per-lesion localization, CE-MRAs detected 18 matched lesions out of 23 lesions (78.3%).

**Table 2:** Combined low-dose time-resolved and single-phase high-resolution contrast-enhanced MR angiography for the diagnosis of spinal vascular diseases.

Disease detection	Digital subtraction angiography		
	Negative	Positive	Total
**Initial evaluation**			
Contrast-enhanced magnetic resonance angiography			
Negative	3	1	4
Positive	0	14	14
Total	3	15	18
**Post-treatment follow-up**			
Contrast-enhanced magnetic resonance angiography			
Negative	5	1	6
Positive	0	0	0
Total	5	1	6

Data presented as n

**Table 3:** Combined low-dose time-resolved and single-phase high-resolution contrast-enhanced MR angiography for the initial characterization of spinal vascular disease.

Initial	no lesion	scAVM	pmAVF	dAVF	ExSpAVM	Total
no lesion	3	0	0	0	1	4 (22%)
scAVM	0	2	1	0	0	3 (17%)
pmAVF	0	0	3	0	0	3 (17%)
dAVF	0	0	0	8	0	8 (44%)
ExSpAVM	0	0	0	0	0	0 (0%)
Total	3	2	4	8	1	18 (100%)

Data presented as n, Parenthesis as percentage (%). Abbreviations: dAVF: dural arteriovenous fistula, scAVM: spinal cord arteriovenous malformation, pmAVF: perimedullary arteriovenous fistula, ExSpAVM: extraspinal arteriovenous malformation

For the post-treatment follow-ups, MRA results were all normal (6 out of 6), while DSA evaluations confirmed that one patient had residual lesion (case 10, same patient with case 9) after partial embolization of extradural AVM using glue, and rest of the subjects were normal. The sensitivity (50.0%; 95% CI: 1.2%–98.7%), specificity (100%; 95% CI: 47.8%–100.0%), and accuracy (85.7%; 95% CI: 42.1%–99.6%) were acceptable considering small sample sizes (n = 6). However, when post-treatment follow-up MRAs were combined with initial MRA, the overall MRA still demonstrated excellent sensitivity (87.5%; 95% CI: 61.6%–98.5%), specificity (100%; 95% CI: 63.1%–100.0%) and accuracy (91.7%; 95% CI: 73.0%–98.9%) in detection of spinal vascular disease.

## Discussion

The spinal axis is too long to be entirely covered by a single FOV during acquisition of MRA. Therefore, several authors have used dual acquisition of overlapped slabs [6], or repeated examinations to focus the shunt site [8, 9]. Ali et al. [6] used two overlapping acquisitions of TR CE-MRA to cover the entire spinal axis with injection of a relatively large total amount of contrast agent. Our protocol with combined low-dose (0.03 mmol/kg) injection TR CE-MRA and single-phase HR CE-MRA in a single session was useful to visualize the lesion without repeated examination and with small amount of contrast media. MRA parameters and the amount of intravenously injected contrast media in our series are summarized with comparison to previous reports in Table 4.

**Table 4:** Magnetic resonance angiography sequences and contrast media for the evaluation of a spinal vascular disease in the present series and previous reports.

Author [reference], year	Case No. (spinal AVM & spinal DAVF)	Field strength	Coverage	Spatial resolution (mm^3^)	Temporal resolution (s)	Dynamic phases	Contrast media
Present series*	16 (13)	3T	280 mm	0.9 × 1.7 × 1.2* 0.7 × 1.0 × 0.5**	1.4* 76**	Multiple* Single**	0.03 mmol/kg* 0.1 mmol/kg**
Binkert [7], 1999	12 (9)	1.5T	280 mm	Not reported	24	Single	0.2 mmol/kg
Farb [8], 2002	9 (9)	1.5T	360 mm	1.0 × 1.0 × 1.2	118	Single	30 cc
Luetmer [9] **, 2005	31 (22)	1.5T	320 mm	1.09 × 1.25 × 1.4	49	Single	52 cc
Mull [16], 2007	34 (31)	1.5T	500 mm	0.9 × 0.9 × 1.2	46–40*	Two	45 cc
Ali [6] ***, 2007	11 (5)	1.5T	Variable	1.2 × 0.7 × 1.3	2.32–6.75	Multiple	30 cc
Vargas [13] ****, 2010	17 (7)	3T	380 mm	1.2 × 1.0 × 0.9	60	Three	0.2 mmol/kg

*Low-dose time-resolved contrast-enhanced magnetic resonance angiography (CE-MRA) followed by single-phase high-resolution CE-MRA.

**Test bolus sequence followed by single-phase CE-MRA.

***Acquisition of two overlapping slabs of multiphasic CE-MRA to cover the entire spinal axis with a variable field-of-view.

****Arterial- and venous-phase MRAs, and delayed high-resolution MR with injection of Vasovist after a test bolus sequence.

There are additional advantages of TR CE-MRA combined with single-phase HR CE-MRA [6, 11, 14, 15]. TR CE-MRA can be performed with a small dose of contrast media, and can also be used as a test bolus sequence to ensure timely bolus arrival during acquisition of the following single-phase HR CE-MRA. Although spatial resolutions and details of small vessel architectures are inevitably lower than conventional CE-MRA, TR CE-MRA has better temporal resolution and effectively demonstrates dynamic information even using low-dose of contrast media. Subsequent single-phase HR CE-MRA, with injection of 0.1 mmol/kg of contrast agent in our series, yielded correct diagnosis in most of the patients with a spinal vascular disease, with reasonably high accuracy which were comparable to previous reports [6, 9, 11, 13, 17, 18]. Thus, in our protocol, combination of low-dose TR CE-MRA and single-phase HR CE-MRA was feasible and effective for pre-angiographic evaluation of spinal vascular diseases.

In initial characterization of spinal vascular disease, CE-MRA in our series showed matched disease characterizations in 86.7% of patients with spinal vascular disease, and in 88.9% of patient when patients without spinal vascular disease were included. Because of complexity of their diagnosis, several efforts have been made and proposed as classification of spinal vascular disease or malformation [19–21]. For the evaluation of diagnostic efficacy of combined low-dose TR CE-MRA and HR CE-MRA in comparison with DSA in patients with spinal vascular disease in our series, we had to exclude spinal vascular tumors, and simplify the classification by the axial localization of the vascular lesion; intramedullary spinal cord AVM, perimedullary AVF, dural AVF, and extraspinal AVM. The diagnosis of spinal dural AVF was made on the basis of an enlarged and tortuous perimedullary vein together with prominent radicular artery in intervertebral foramen [7, 8, 22]. CE-MRA showed matched characterization of all 8 patients with spinal dural AVF in our series. When arteriovenous shunt was seen on the surface of spinal cord, perimedullary AVF was suggested. The diagnosis of spinal cord AVM was made when dilated nidus was seen inside the short segment of spinal cord with dilated arteries and veins. In our series, CE-MRA resulted in matched suggestion of 3 of 4 patients with perimedullary AVF, 2 of 2 patients with spinal cord AVM, and unmatched suggestion of spinal cord AVM in a patient with perimedullary AVF. In this patient with unmatched suggestion, markedly dilated venous sac with compression of spinal cord on CE-MRA made false characterization. However, in our series, CE-MRA yielded effective differentiation between the dural and intradural vascular lesions, which was also valuable pre-angiographic information. Meanwhile, extraspinal AVM with small single spinal intradural venous drainage was not identified on CE-MRA. Because the single intradural draining vein in this patient was too small to be seen on low-dose TR CE-MRA, nidus of this AVM was located caudally outside the FOV of subsequent HR CE-MRA.

Previous studies conducted by Luetmer et al., demonstrated 92.8% of detecting feeders within 1 vertebral level [10], and studies conducted by Mull et al., demonstrated about 73.6% of detecting the feeders within 1 vertebral level [11]. In our series, CE-MRA showed matched per-case localization of arterial feeders within 1 vertebral level in 80%, and it was 78.3% for the per-lesion localization. Our findings demonstrated similar or as effective localization of feeders compared with previous results.

Moreover, single-phase HR CE-MRA using our protocol was of higher resolution compared with previous reports, and the total amount (0.13 mmol/kg) of injected contrast agent was smaller [6–9, 13, 16, 23]. Spinal arteries and veins are very small; therefore, the spatial resolution of spinal cord MRA should be sufficient for their visualization [23]. According to the information provided by low-dose TR CE-MRA, more focused single-phase HR CE-MRA with a smaller FOV and higher resolution may further improve the diagnostic efficacy for smaller additional arterial feeders, although this hypothesis should be validated in future studies.

In addition to proper diagnosis, characterization and localization of spinal vascular lesions, spinal CE-MRA is also useful in pre-angiographic evaluation by accurately and three-dimensionally depicting the sites of origin of the intercostal and lumbar arteries from the aorta. Understanding the 3D relationship between the initial segment of segmental arteries with reference to the aorta aids subsequent catheter angiography procedures with a reduced radiation dose and volume of contrast agent associated with angiography [9]. In our experience, axial segmented MIP reconstruction of each vertebral level was especially helpful in identifying the sites of origin of intercostal and lumbar arteries from the aorta, which resulted in easy and timely catheterization of each intercostal and lumbar artery during subsequent diagnostic spinal DSA. Pre-angiographic CE-MRA is also useful in cases with segmentally hypoplastic or aplastic intercostal vessels with collateral flow from the adjacent level below or above (Fig 2), which also facilitates planning of the subsequent diagnostic spinal DSA.

In our patients with dural AVFs, 3D single-phase HR CE-MRA was mostly unable to depict the normal anterior and posterior spinal arteries. Nijenhuis et al. reported a 97% success rate for localization of the Adamkiewicz artery by CE-MRA in patients with a thoracoabdominal aortic aneurysm [23, 24]. In their reports, 0.3 mmol/kg gadolinium-based contrast media was intravenously injected to obtain two phases of HR MRA with a 0.8 × 0.8 × 1.2 mm voxel size at 1.5 T. In contrast, single-phase HR CE-MRA in our series was obtained with injection of 0.1 mmol/kg contrast media, and spatial resolution was worse than their report. Because larger number of the patients in our series had vascular disease with dilated intrathecal vessels, dilated perimedullary vessels can also complicate identification of small normal anterior or posterior spinal arteries. However, the purpose of spinal CE-MRA in our protocol was to detect and characterize spinal vascular disease as in pre-angiographic imaging; HR CE-MRA with injection of 0.1 mmol/kg seemed to be reasonable for this purpose.

Advances in MR sequences and technologies have enabled visualization of spinal vascular lesions in a non-invasive manner and with high accuracy before DSA evaluation. There are multiple MR imaging studies that have evaluated spinal vascular lesions using newly developed sequences and techniques. One recent example was the study by Kralik et al. [25], which used sagittal 3D volumetric T2-weighted imaging (T2WI) sequences (constructive interference in steady state, fast imaging employing steady-state acquisition, SPACE). Taking advantage of visualizing pathologically engorged vessels on T2WI is not only sensitive in terms of detecting the flow void, but also avoids unnecessary contrast media injection. In addition, spinal cord edema can be also appreciated simultaneously because of the fluid sensitive sequence.

This retrospective study had several limitations, the first of which was the small sample size (n = 17) in the evaluation of large variety of spinal vascular disease. Nevertheless, our study demonstrated high diagnostic performance in terms of pre-angiographic detection and characterization of arteriovenous shunts and localization of feeding arteries after combined acquisition of low-dose TR CE-MRA followed by single-phase HR CE-MRA. CE-MRA after treatment of spinal vascular disease was not compared with DSA in our series. Secondly, there were very small number of patient on follow-up after the treatment was included, thus efficacy could be independently evaluated. The effectiveness of out protocol in the evaluation of post-treatment status should be further validated in comparison with DSA. Third, the verification via surgical excision nor pathology confirm was not conducted.

## Conclusions

Spinal CE-MRA with combined acquisition of low-dose TR CE-MRA followed by single-phase HR CE-MRA at 3T was effective and accurate as pre-angiographic evaluation of spinal vascular diseases. Low-dose TR CE-MRA not only provided information regarding detection of bolus arrival, but also helped proper placement of FOV of subsequent HR CE-MRA.

## Supporting information

S1 Table Summary of imaging findings in patients with spinal vascular disease. * 0: negative, 1: positive, ** 0: No lesion, 1: Spinal cord arteriovenous malformation, 2: Perimedullary arteriovenous fistula, 3: Spinal dural arteriovenous fistula, 4: Extraspinal arteriovenous malformation.
(PDF)
