# Supplementary material for: Diagnostic accuracy and efficiency of combined acquisition of low-dose time-resolved and single-phase high-resolution contrast-enhanced magnetic resonance angiography in a single session for pre-angiographic evaluation of spinal vascular disease
Source: PLoS One. 2019 Mar 28;14(3):e0214289. doi: 10.1371/journal.pone.0214289 (PMC6438605; doi:10.1371/journal.pone.0214289)
Supplement: S1 Table — * 0: negative, 1: positive, ** 0: No lesion, 1: Spinal cord arteriovenous malformation, 2: Perimedullary arteriovenous fistula, 3: Spinal dural arteriovenous fistula, 4: Extraspinal arteriovenous malformation. (PDF) [file pone.0214289.s001.pdf]

Patient	Case	Initial or follow-up study	DSA finding*	type on DSA**	Level of disease on DSA	Low-dose Time-resolved MRA finding*	Single phase high resolution MRA finding*	Type on MRA**	Level of disease on MRA	Treatment
1	1	Initial	1	3	Lt T6	1	1	3	Lt T6	Embo
2	2	Initial	1	2	Lt T8	0	1	1	T9	Cyberknife
3	3	FU	0	0	0	0	0	0		Surgery
4	4	Initial	1	3	Lt Int Iliac	1	1	3	Int Iliac	Embo
3	5	Initial	1	4	Rt T12	1	1	4	Rt T12	Surgery
5	6	Initial	1	1	Rt T10, Lt T10, Lt T9	1	1	1	Rt T10, Lt T10, Lt T9	Surgery
6	7	Initial	1	2	Lt L3	1	1	2	Lt L3	Surgery
7	8	Initial	1	3	Rt L1	1	1	3	Rt L1	Embo/Surgery
8	9	Initial	1	4	Lt L4	0	0	0		Embo
8	10	FU	1	3	Lt L4	0	0	0		
9	11	Initial	1	1	Lt T9	1	1	1	Lt T9	
10	12	Initial	1	2	Rt T12, B T8, Lt L1, Rt L2, Lt L1	1	1	2	B T8	
10	13	FU	0	0		0	0	0		Surgery
11	14	Initial	0	0		0	0	0		
12	15	Initial	1	3	Lt T5	1	1	3	Lt T5	Surgery
13	16	Initial	1	2	Lt L1, Rt T10	1	1	2	LT L1, Lt L2/3	Surgery
12	17	FU	0	0		0	0	0		
13	18	FU	0	0	0	0	0	0		
14	19	Initial	1	3	Rt T12, Lt L1	1	1	3	Rt T12, Lt L1	
5	20	Re_Initial	1	3	Rt S2/S3	1	1	3	Rt S2	Surgery
15	21	Initial	0	0	0	0	0	0	0	
12	22	FU	0	0		0	0	0		
16	23	Initial	0	0	0	0	0	0	0	0
17	24	Initial	1	3	Rt T6	1	1	3	Rt T6	Surgery
